# Effectiveness of Passive and Active Surveillance for Early Detection of SARS-CoV-2 in Mink during the 2020 Outbreak in the Netherlands

**DOI:** 10.1155/2024/4793475

**Published:** 2024-04-12

**Authors:** Inge M. G. A. Santman-Berends, Gerdien van Schaik, Marieke Augustijn-Schretlen, Irene P. I. H. Bisschop, Jan de Rond, Paola A. Meijer, Harold M. J. F. van der Heijden, Francisca C. Velkers, Marion P. G. Koopmans, Wim H. M. van der Poel, Lidwien A. M. Smit, Arjan J. A. Stegeman, Reina S. Sikkema, Bas B. Oude Munnink, Renate W. Hakze-van der Honing, Robert-Jan Molenaar

**Affiliations:** ^1^Department of Research and Development, Royal GD, Deventer, Netherlands; ^2^Department of Population Health Sciences, Faculty of Veterinary Medicine, Utrecht University, Utrecht, Netherlands; ^3^Department of Poultry Health, Royal GD, Deventer, Netherlands; ^4^Department of Viroscience, Erasmus MC, Rotterdam, Netherlands; ^5^Wageningen Bioveterinary Research, Lelystad, Netherlands; ^6^Institute for Risk Assessment Sciences (IRAS), Utrecht University, Utrecht, Netherlands

## Abstract

Starting December 2019, a novel coronavirus (SARS-CoV-2) spread among humans across the world. From 2020 onward, farmed mink were found susceptible to the virus. In this paper, we describe the Dutch surveillance system and the added surveillance components for early detection of SARS-CoV-2 outbreaks and their results in Dutch mink farms. In the Netherlands, a surveillance system was in place in which mink farmers could submit carcasses for postmortem evaluation and could contact a telephone helpdesk for veterinary advise. Through this system, the first SARS-CoV-2 outbreak in two mink farms was detected in April 2020. Immediately, the Dutch Ministry of Agriculture commissioned a consortium of statutory and research institutes to intensify the surveillance system. The program consisted of both passive surveillance, i.e., mandatory notifications and active surveillance components, i.e., serological screenings and weekly risk-based sampling of dead mink for early detection of new SARS-CoV-2 infections. When one of the surveillance components indicated a suspicion of a possible SARS-CoV-2 infection, follow-up samplings were conducted and at confirmation, all mink were culled. During 2020, 67 out of 124 mink farms that were under surveillance became infected with SARS-CoV-2 (54%). Of these, 31 were detected based on clinical signs (passive surveillance of clinical signs) and 36 were detected through active surveillance. From the mink farms with a new SARS-CoV-2 outbreak that was detected through the surveillance, in 19% of the farms (*n* = 7), the mink never showed any clinical signs of SARS-CoV-2 and might have been missed by the passive notification system. This study underlines the added value of a surveillance system that can quickly be intensified. The subsequent combination of both passive and active surveillance has shown to be effective in the early detection of emerging pathogens, which is important to minimize the risk of zoonotic spill-over.

## 1. Introduction

In December 2019, a novel coronavirus (SARS-CoV-2) was identified as the cause of a viral pneumonia outbreak in China [1]. Since then, SARS-CoV-2 has spread rapidly, resulting in an ongoing worldwide pandemic [2].

At the start of the pandemic, the susceptibility of different animal species, including mink, for SARS-CoV-2 was still unknown. However, during the pandemic, it became apparent that a large number of animal species were susceptible to SARS-CoV-2 infections including dogs, cats, ferrets, mink, hamsters, white-tailed deer, and monkeys [3–5] and that in a number of animal species, the virus could also easily spread [6].

Before 2020, Europe was the leading continent in mink farming with the production of over 34 million mink skins in approximately 2,750 mink farms in 22 countries, accounting for 58% of total global mink production in 2018 [7]. The leading mink producing European countries included Denmark (>1,100 farms), Poland (>300 mink farms), and the Netherlands (>100 mink farms) [8, 9]. However, after outbreaks of SARS-CoV-2 in mink farms in the Netherlands in early 2020 and later on also in several other countries, measures were taken for early detection of new SARS-CoV-2 infections in farmed mink and to cull mink farms that tested positive [3, 10]. Later on, full-length virus genome sequencing revealed that the SARS-CoV-2 variants that were found in mink were also detected in the farm owners and workers, in cats, in environmental samples, and in the local community [9, 11–14]. Based on these results, it was concluded that farmed mink constitute a potential virus reservoir challenging pandemic control, supporting the chosen strategy to control SARS-CoV-2 infections in mink by culling.

In the Netherlands, since 2002, an animal health surveillance system with, amongst others, a helpdesk is in place for farmers and veterinarians who have questions related to signs of disease in their livestock [15]. As part of this system, in 2012, an additional surveillance component was implemented for mink farmers and veterinarians in which they could submit carcasses of mink for a subsidized postmortem evaluation. The first cases of SARS-CoV-2 in two mink farms were detected through this surveillance component in April 2020 after Royal GD was consulted about outbreaks of clinical respiratory disease and mortality in mink with unknown cause [16]. These findings were in line with the outcomes of a national zoonosis meeting that stated that mink could potentially be sensitive for SARS-CoV-2 infection [10]. Immediately, a consortium of statutory and research institutes was commissioned by the Dutch Ministry of Agriculture, Nature and Food Quality and additional active surveillance activities were initiated targeting both mink and mink handlers (farmers and personnel) to ensure early detection of new infections [17]. Despite the increased awareness, the virus spread rapidly within and between mink farms, and from June 2020 infected mink farms had to be culled. Later that year, the Netherlands decided to speed up the intended ban on mink farming from 2024 to early January 2021 [18]. Although a ban on mink farming has since been implemented in other countries as well (e.g. Norway, United Kingdom, Italy), culling is not mandatory and mink farming remains a large industry in many countries worldwide stressing the importance of effective monitoring systems for early detection of disease outbreaks such as SARS-CoV-2 [8].

In this paper, we describe the surveillance system in place in and before 2020 for early detection of new SARS-CoV-2 infections. Furthermore, the added value of active surveillance compared to passive surveillance based on notification of clinical signs is described.

## 2. Materials and Methods

### 2.1. Population

**Figure 1:**
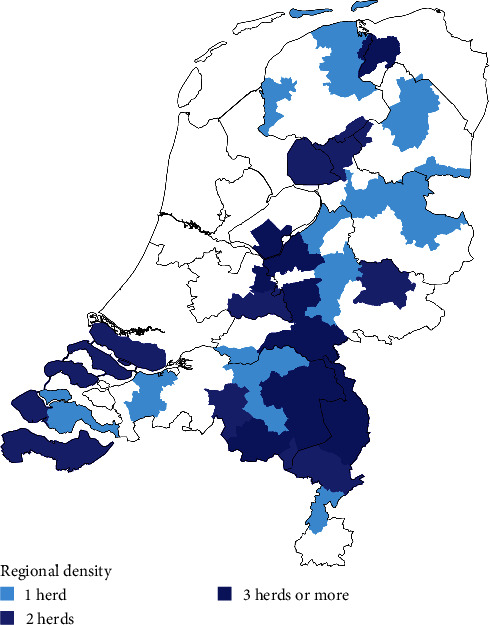
Density map of Dutch mink farms per two-digit postal code area at the start of the SARS-CoV-2 pandemic in May 2020.

All mink farms in the Netherlands were under SARS-CoV-2 surveillance and were included in this study. At the start of 2020, before the SARS-CoV-2 pandemic, 126 mink farms were present in the Netherlands. These farms were mainly located in the south-eastern part and the central part of the country (Figure 1). The average farm had 5,165 female mink (median 4,535, 5–95^th^ percentile: 458–11,800). In the Netherlands, there was one reproductive cycle per year. The breeding of mink occurred in March and pups were born in May of each year. After all pups were born, on average 32 thousand mink per farm were present (median 27,671, 5–95^th^ percentile 1,000–79,355) in 2020.

### 2.2. Surveillance System in Livestock

**Figure 2:**
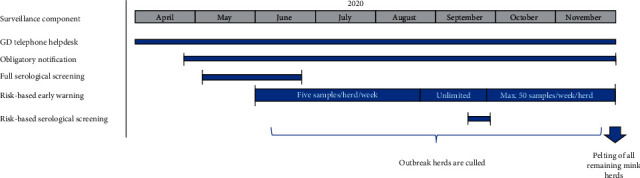
Schematic overview of the surveillance components for SARS-CoV-2 in mink farms in the Netherlands between April and November 2020.

The Dutch surveillance system consists of multiple active and passive surveillance components tailored to the different livestock sectors [15]. For mink, the surveillance system included the possibility to submit carcasses for subsidized postmortem investigation, a telephone helpdesk that farmers could contact with questions related to the health of their mink, case detection of endemic diseases by regular testing of fallen stock on a number of representative mink farms, frequent meetings with national and European mink veterinarians in the Netherlands to discuss recent disease outbreaks and horizon scanning. During 2020, the existing surveillance activities for mink were expanded rapidly to include additional components for the early detection of new cases, which were implemented at different time points and for different durations depending on the aim of each component (Figure 2). The existing and added surveillance components are described in more detail below.

#### 2.2.1. GD Helpdesk and Postmortem Facilities

As part of the mink health surveillance system at GD a helpdesk “Veekijker” is available which can be phoned free of charge. This helpdesk is consulted approximately 9,000 times per year across species for which the helpdesk is in place and is operated by veterinary specialists that are available for consulting when questions arise related to the health of livestock [15]. For different livestock species, different veterinary specialists are available. The unique herd number and the topic of the phone calls are registered in a database combined with diagnostic or postmortem results if available. Outbreaks of emerging zoonotic pathogens, such as SARS-CoV-2, are reported and discussed within the Dutch Zoonosis Structure [19], which is an integrated human–veterinary collaboration for risk analysis for emerging zoonosis. In this collaboration, signals of emerging zoonosis are shared, assessed and, when necessary, control measures are taken in an integrated “One Health” approach. When, in 2020, postmortem evaluations of dead mink in combination with information received via the helpdesk indicated that clinical signs in mink could be related to SARS-CoV-2 infections in mink, the NVWA was immediately contacted and the testing protocol of the competent authority was implemented, as described below.

#### 2.2.2. Obligatory Notification System

The obligatory passive surveillance system came into force on 26 April 2020. Mink farmers were obliged to notify all clinical signs indicative for a possible SARS-CoV-2 infection to the competent authority of the Dutch Food and Consumer Product Safety Authority (NVWA). Signs that could be associated with SARS-CoV-2 infections were related to the respiratory tract, e.g., watery discharge, tachypnea, accessory breathing, excessive lacrimation, and breathing sounds like coughing and sneezing. In addition, signs of general disease, such as apathy, reduced feed intake and increased mortality were notifiable as well [20]. When farmers were notified clinical signs, a subsequent testing protocol was implemented, which is described in Section 2.2.6.

#### 2.2.3. Full Serological Screening

The first active surveillance component that was implemented was a screening of the presence of SARS-CoV-2 antibodies in all mink farms using an in-house SARS-CoV-2 ELISA of GD [21] and was conducted in the period between 5 May and 23 June 2020. Mink farms were sampled by a GD veterinarian, who collected blood samples from clipped toenails of 60 randomly selected mink per farm using filter coombs. Based on 60 samples, presence of SARS-CoV-2 antibodies in the mink farm could be detected with 95% confidence under the assumption that if the virus was present, at least 10% of the mink would have seroconverted (see also Section 2.3). If no antibodies against SARS-CoV-2 were detected, the farm was considered negative and entered the risk-based real-time polymerase chain reaction (RT-PCR) surveillance of weekly testing of dead mink. When antibodies were detected in up to three samples (either one, two, or three), these were retested (see Section 2.3). When all samples during this retest were negative, the farm was considered negative and entered the risk-based surveillance. In all other cases (i.e., 1–3 positive results after retesting or more than three positive samples in the initial test), the testing protocol that is described in Section 2.2.6 was implemented.

#### 2.2.4. Risk-Based RT-PCR Testing of Dead Mink

This risk-based early warning surveillance component was considered the second active surveillance component. This consisted of RT-PCR-based SARS-CoV-2 screening in dead mink, which was implemented from June 2020. Dead mink were considered at higher risk of having had a SARS-CoV-2 infection than the other mink on the farm. On a weekly basis, mink farmers were obliged to submit (a selection of) dead mink to GD for investigation. Between June and August, farmers were requested to submit up to a maximum number of five dead mink per week. Because the outbreaks in mink farms could not be prevented, it was advised by the Outbreak Management Team Zoonoses to the government to enhance the early warning system in mink in order to detect outbreaks at an early stage and subsequently cull infected farms. Given the limited statistical power of a sample of five and the importance of early detection, at the end of August, farmers were obliged to submit all dead mink for investigation. Because subsequently, the number of submitted mink surpassed the pathological capacity in the Netherlands and because of increasing evidence that when introduced, SARS-CoV-2 spread rapidly through the herd, from late September on farmers had to submit up to a maximum of 50 dead mink per week allowing for detection of a within-herd prevalence of 15% with a precision of 10% and 95% confidence. From all submitted mink, throat samples were taken and pooled per five animals. The pooled samples were tested using a RT-PCR and when positive animals were detected, the farm was classified as suspect and the testing protocol for suspected mink farms was implemented.

#### 2.2.5. Risk-Based Serological Screening

This active surveillance component was introduced in September 2020 and consisted of a second serological screening in the high-risk area. This high-risk area was defined as the region with the highest mink farm density in the Netherlands and where mink farms had been culled in the previous months due to SARS-CoV-2 outbreaks. The farms that entered this surveillance scheme had tested negative in the risk-based surveillance on dead mink up to September. This second screening was conducted in a similar manner as the initial full serological screening conducted at the start of the SARS-CoV-2 epidemic in mink in the Netherlands.

#### 2.2.6. Testing Protocol in Suspected Mink Farms

When one of the surveillance components indicated a suspicion of a possible SARS-CoV-2 infection, the farm was visited by a team of veterinarians from the NVWA and GD for a clinical inspection and official sampling [20]. During these visits, all clinical signs of SARS-CoV-2 in mink were recorded. Additionally, throat and rectal swabs were taken from 20 selected high-risk mink (i.e., if present, with clinical signs, and otherwise samples were taken evenly spread throughout the farm) for further investigation. The submitted samples were tested for presence of virus using a RT-PCR. When at least one of the samples resulted in a positive result, the farm was classified as being infected and all mink were culled by the NVWA as soon as possible.

### 2.3. Diagnostic Tests

For the mandatory serological screening of mink, blood collected and dried on paper filter coombs, was eluted with an in-house assay buffer. This corresponded with approximately 2 *µ*L of serum input. The eluted samples were tested for SARS-CoV-2 antibodies using an in-house indirect ELISA using a recombinant protein for the receptor-binding domain (RBD) of the S1 spike protein of SARS-CoV2. When antibodies were detected in up to three samples, these samples were retested in the ELISA with a recombinant (full) S1 protein instead of the recombinant S1-RBD protein as coating antigen. The ELISA used an S/P-ratio of 0.20 as cut-off value and had a herd specificity of 94.2% and a herd sensitivity of 91.8% assuming a within-farm prevalence of at least 10% with 95% confidence, and considering a herd positive when at least one out of 60 samples tested positive [21].

For the risk-based early warning, the same RT-PCR test, which detected the presence of viral RNA coding for the SARS-CoV-2 E gene [22] was used by GD and also for confirmation of suspicion by the Dutch reference laboratory (WBVR). Virus-positive samples were subjected to full genome sequencing for confirmation [14].

### 2.4. Available Data

Data on the location (postal code) of the farm and the farm size for all mink farms in the Netherlands was available from the Dutch enterprise agency in Assen (RVO). Additionally, three datasets were available with the diagnostic results of the different surveillance components and the infected farm investigation. The datasets and the parameters are presented in Table 1. Although detailed information on the outbreaks, including the type and severity of clinical signs, was available, we focussed on the detection of new outbreaks. More detailed results of the transmission of the virus between farms and the clinical signs of SARS-CoV-2 in mink during an outbreak have been described in Wolters et al. [20].

### 2.5. Data Analysis

The available datasets were combined and analyzed using Stata® version 15.1 [23]. Results were summarized using summary statistics and frequency tables and are presented graphically where possible. The SPMAP procedure in Stata® was used to map the results per two-digit postal code in the Netherlands. The number of SARS-CoV-2 infected mink farms that were detected by each of the surveillance component and the moment of detection is described. The cumulative incidence of detected farms relative to the number of active mink farms was calculated and graphically presented on a monthly basis. Finally, detection of SARS-CoV-2 outbreaks in mink farms by the notification system and the additional surveillance components (serological screening and risk-based early warning) were compared. All mink of farms that were detected through the surveillance components were subsequently culled within days. In some farms, clinical signs became visible between the date of detection and the date of culling. In these cases, the number of days between the moment of detection and observation of the first clinical signs (if any) was evaluated and described.

## 3. Results

In the Netherlands, 126 mink farms were present at the start of 2020. After the two initial SARS-CoV-2 positive mink herds that were detected in April, the remaining 124 mink farms entered the intensified surveillance system. From these 124 mink farms, two farmers notified clinical signs of SARS-CoV-2 to the authorities in May 2020 and the infection was confirmed. The remaining 122 farms were screened for SARS-CoV-2 antibodies in May and June 2020 and entered the risk-based surveillance scheme.

### 3.1. First Serological Screening of 122 Mink Farms

The number of sampled mink per farm varied between 59 and 65 mink. In one farm, a double sample size of 118 mink was accidentally conducted. In 113 out of 122 mink farms, none of the tested mink were antibody positive. In the nine farms where antibodies were found, six farms antibodies were detected in up to three mink and in the remaining three farms larger numbers of seropositive mink were detected (*n* = 6, 21, and 24 mink with antibodies).

Seven out of the nine farms on which antibody positive mink were found were located in the south-eastern part of the Netherlands where the mink farm density was highest (Figure 1). Five out of these seven farms reported clinical signs within days after the farms were detected as being antibody positive. In the other two farms, in, respectively, one and two out of the 60 sampled mink antibodies were detected during the serological evaluation that was conducted in these farms at the end of May, and at that time, results were not confirmed in the RT-PCR test. In the risk-based surveillance that was subsequently implemented, these farms tested RT-PCR positive in June and the mink started to show clinical signs, respectively, 2 days before and 6 days after the confirmatory results became available.

The mink on two farms in which antibodies were detected (in two mink per farm) that were outside the prementioned region, did not show any clinical signs, and the serological results of these farms were not confirmed by RT-PCR. Eventually, in both farms, neither virus nor antibodies were detected throughout the surveillance period (until November 2020) when all mink were slaughtered and pelted.

The time between the serological results and the observation of the first clinical signs varied. In one farm, clinical signs were already noticed by the farmer 9 days prior to the serological screening. In the other four farms, SARS-CoV-2 was first detected in the serological screening, and subsequently clinical signs were observed during an official visit between 0 and 3 days later. The clinical signs that were recorded most frequently by the official veterinarians included a watery discharge from nose and eyes, an abnormal respiration and shortness of breath, decreased feed intake, and increased mortality and apathy [20]. All five farms were culled after the infection was confirmed which was between 2 and 10 days after the first clinical signs were evident (mean and median 6 days).

### 3.2. Risk-Based Surveillance on Dead Mink

From June 2020, all mink farms that were still SARS-CoV-2 negative entered the obligatory early warning system in which dead mink had to be submitted to GD for RT-PCR SARS-CoV-2 screening. The number of submitted dead mink per farm varied from a median of 3–15 per week (Table 2). The number of farms that participated in the risk-based surveillance decreased in time due to culling efforts.

In June, 2.7% of the mink farms SARS-CoV-2 positive mink were found (three out of 111). Up to August, the percentage of farms in which virus was detected increased to 19.4% (20 out of 103). Thereafter, both the number of active mink farms and the number of farms in which virus was detected decreased rapidly (Table 2). When SARS-CoV-2 was detected in a mink farm, on average 49% of the submitted mink carcasses tested positive.

### 3.3. Second Serological Screening of Mink Farms in the High-Risk Area

In September 2020, 21 mink farms without indication of a SARS-CoV-2 outbreak were still active in the geographic area with most mink farms (Figure 1). Between 15 and 30 September, the second serological screening was conducted in these farms, resulting in 1,282 results from 21 different mink farms (on average 61 samples were submitted per farm). In three mink farms, antibodies against SARS-CoV-2 were found. All three farms were located in a different postal code area. SARS-CoV-2 infections on two out of three farms were confirmed by RT-PCR and the mink started to show clinical signs, respectively, 1 and 7 days after detection in the serological screening. Both mink farms were culled at, respectively, 5 and 10 days after initial serological detection. A SARS-CoV-2 infection in the third farm could initially not be confirmed and none of the mink showed clinical signs at the time. However, in October, mink started to show clinical signs indicating SARS-CoV-2 infection, and the farm was culled on 17 October 2020.

### 3.4. Comparison of the Official Notifications with the Additional Surveillance Components

**Figure 3:**
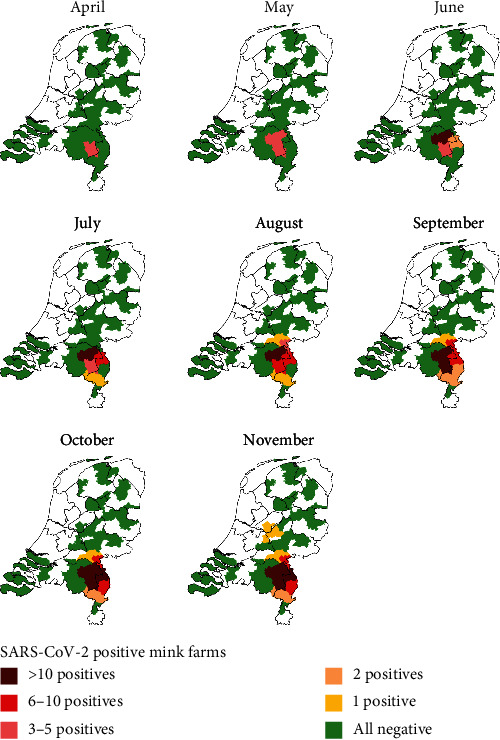
Cumulative incidence of SARS-CoV-2 in Dutch mink farms per two-digit postal code area between April and November 2020.

Of the 126 Dutch mink farms that were active at the beginning of 2020, four farms notified clinical signs related to SARS-CoV-2 and were confirmed positive before initiation of the surveillance system [16, 24]. From the 122 farms that were placed under additional surveillance, 65 farms became infected with SARS-CoV-2. Of these, 36 were first detected in either the risk-based surveillance, serological screening, or because of subsequent tracking and tracing of contact farms (*n* = 2 farms) and 29 were detected based on occurrence of clinical signs. Most infections occurred in the south-eastern part of the Netherlands (Figure 3).

In the majority of the 36 farms that were detected through the risk-based surveillance or serological screening, clinical signs became apparent within days after detection. However, in seven farms (19%, 95% CI: 8%–36%), clinical signs were never observed and these farms may not have been detected without the additional active surveillance. Farms that were detected in either the risk-based surveillance or serological screening were notified to the authorities and the confirmation was obtained on average 3 days later (median 3 days, min. 0, and max. 21 days). The duration of 21 days was an exception and was a small mink farm in which antibodies were found in the second screening which could initially not be confirmed by RT-PCR.

## 4. Discussion

This paper describes the passive and active surveillance components for early detection of SARS-CoV-2 infections in Dutch mink farms. The system in place was able to detect SARS-CoV-2 infected mink farms at an early stage. However, the surveillance activities and consequent containment measures were not able to stop the spread of SARS-CoV-2 in mink in the Netherlands. There can be several reasons for the inability to stop transmission of SARS-CoV-2 between mink farms. One reason could be that detection in mink was not fast enough and containment measures came too late, which allowed for spread in the high-risk period of an undetected infection. Other reasons could be that the contact structure between the farms could not be adjusted sufficiently to reduce the transmission between farms or the fact that the virus was not only transmittable between mink but also from infected humans to mink. During the study period in 2020, there was no preventive screening of SARS-CoV-2 in humans that were in contact with mink given the limited test capacity in the spring of 2020. Implemented regulations only stated that farmers and farm workers had to be tested when they showed clinical signs indicative for a SARS-CoV-2 infection or when they were exposed to the virus because SARS-CoV-2 was found in the mink. In the latter case, the farmer and farm workers had to be tested at day 3 and day 10 after detection [14, 25]. Furthermore, additional regulations regarding biosecurity in mink farms were implemented as well as restrictions for farm workers, for whom it was no longer allowed to work on multiple mink farms.

Over half of the infected farms were detected in the active risk-based mink surveillance before clinical signs, as described by Oreshkova et al. [16], which were reported to the authorities. In case of emergence of a zoonotic pathogen such as SARS-CoV-2, early detection is of utmost importance to reduce the risk of the pathogen spreading between farms and from animals to humans and vice-versa. Therefore, in this specific case, the active surveillance had added value in the early detection of the virus in addition to passive mandatory notifications based on detection of clinical signs. Nevertheless, it may be that part of these farms would have been detected based on reporting of clinical signs a few days later. This would however have increased the risk of spread to other farms and infection of humans, pets, and potentially wildlife in contact with the mink in the meantime. In the majority of the detected mink farms, clinicals signs were detected at the official visit a couple of days after detection of the virus in the early warning system, but almost one out of five (19%) positive farms had no clinical indication of an outbreak. Given that the SARS-CoV-2 positive farms were culled, it is unknown whether the farms without mink with clinical signs would eventually have shown signs. The percentage of mink farms that showed no clinical indication of SARS-CoV-2 in the Netherlands was slightly lower than what was observed in Denmark where it was found that in one-third of 215 infected mink farms the mink never showed clinical signs [3].

For cost-effectiveness calculations, it would have been interesting to determine the number of days that the infection was detected earlier in the active surveillance components compared to the passive notification system. However, this was not possible given the fact that the results of the surveillance created awareness when positive mink were found and subsequent actions were taken. Therefore, the difference in days between onset of clinical signs and the official confirmation did not greatly differ between the active and passive surveillance components. It is unknown if, and at what moment, the farms would have been detected when the active surveillance components would not have been in place. For most farms, the difference would probably only be a couple of days given the rapid display of the first clinical signs after detection. Because of these limitations of the data and the absence of a control group, a cost-effectiveness calculation could not be conducted. However, cost-effectiveness was of less importance in the emergence of SARS-CoV-2, given that the virus is zoonotic [26], mink appeared to play a role in infecting humans [9] and may act as virus reservoirs increasing the risk of mutations [27]. Therefore, to miss any infected farms was unacceptable, which is the rationale for additional active surveillance that was implemented in several countries with mink farming systems [28, 29].

In the specific case of SARS-CoV-2 in mink, one of the clinical signs that was observed was increased mortality [3, 20, 24]. Therefore, we used a risk-based sampling design for early warning in which dead mink were submitted for SARS-CoV-2 sampling on a weekly basis. An additional advantage of this sampling design was that the farmers could collect the dead mink themselves and that there was no need for the veterinarians or authorities to enter the farm. In that way, indirect transmission of the virus between different farms associated with the sampling process could be avoided. Risk-based sampling of dead mink for SARS-CoV-2 had additional value to passive surveillance for clinical signs. On several farms, the SARS-CoV-2 infection caused hardly any clinical signs and only a few dead mink. A drawback of this sampling design was that the number of sampled mink was limited in the first month (a maximum of 5 per week). This sample size was based on the assumption that if SARS-CoV-2 was present in a farm, a high prevalence in dead mink would be found. However, given that the causes of death can be numerous, at onset of an infection the within-herd prevalence can still be low, and the major importance of rapid detection, from August on the ministry obliged submission of all dead mink for SARS-CoV-2 testing. Later on, the number was limited to at most 50 dead mink per herd for capacity reasons and because sampling a higher number appeared unnecessary given the rapid transmission of the virus within the farms. Changing the study design was a result of the lack of information at the onset of the crisis and both experts and government were constantly changing their decisions based on the newest information available. Our results showed that the combination of passive and active surveillance was effective in the early detection of SARS-CoV-2 in mink farms.

With the serological surveys in which 60 mink were sampled, lower prevalences could be detected, which may be more effective for pathogens that spread slowly in a farm or that only infect a part of the mink on the farm. However, in the case of SARS-CoV-2, several studies have shown that the within herd prevalence in mink farms increased quickly and that a large proportion of the mink on the farm became infected [3, 27]. For SARS-CoV-2, weekly testing of five dead mink is probably more cost-effective to detect new SARS-CoV-2 introductions into mink farms as compared to large-scale serological surveys at a lower frequency. This risk-based testing of dead mink was supported by EFSA who concluded that the most appropriate surveillance approach for rapid detection of SARS-CoV-2 in mink farms is to confirm the farms' infection status based on either a suspicion of SARS-CoV-2 due to clinical manifestation, test results, or increased mortality [30].

During the SARS-CoV-2 pandemic, different countries decided on different surveillance strategies to detect infections in mink. Poland tested for example oral fluids of 20 symptomatic mink per farm [8]. In Denmark, a combination of throat swabs and blood sampling was used for SARS-CoV-2 evaluation on high-risk farms, i.e., farms with symptomatic mink, contact farms of infected mink farms, farms on which humans tested SARS-CoV-2 positive, or farms that were detected through surveillance on dead mink [3]. Less invasive sampling strategies were also suggested as option for SARS-CoV-2 surveillance in mink, such as environmental sampling [31], and virus could be detected in fecal, environmental samples, and in dust samples [12, 32]. However, these novel sampling methods needed further validation which was not feasible during the timeframe of the SARS-CoV-2 outbreaks in 2020 in the Netherlands.

The SARS-CoV-2 epidemic in mink created awareness that at incursion of a new zoonotic disease, one cannot wait until all the knowledge about pathophysiology and epidemiology of the causative pathogen is available. Immediate action is needed when human health is at stake and actions can be adapted as time proceeds and more knowledge is obtained. In the Netherlands, these actions consisted of immediate implementation of mandatory notification of clinical signs, a screening of all farms, followed by continuous surveillance tailored to the specific traits of SARS-CoV-2 virus in mink, as far as was known. The added active surveillance components for early detection of new SARS-CoV-2 infections in mink farms are examples of a surveillance strategy that can be widely implemented for a large number of diseases regardless of the targetted animal species. In addition to surveillance in mink farms, during the SARS-CoV-2 pandemic also the presence of SARS-CoV-2 in other animal species in the Netherlands (e.g., rabbits, cats, and wildlife) [33], spill-over from mink to free-ranging animals [34] and to other (companian) animals present in the farm [11] was investigated.

The steps taken in the case of SARS-CoV-2 in mink in the Netherlands, i.e., early detection in the existing surveillance system, official notification, insight in the status quo, and surveillance with subsequent risk mitigating actions is in all cases a useful strategy to minimize the risk when new diseases emerge in livestock. Similar conclusions were drawn for an emerging disease in Dutch cattle [35] and are substantiated as necessary pandemic preparedness by the WOAH [36] and EFSA [30]. Although in the Netherlands, mink farming has been banned, the lessons learned can be used in other countries where mink farming has been restarted or where the decision was made not to cull infected mink farms but to keep on monitoring these mink farms to prevent further spread.

## 5. Conclusion

This study provided insight in the effectiveness of different surveillance components for early detection of disease. In the case of SARS-CoV-2, the existing passive surveillance was able to detect the virus in mink farms at an early stage. Additionally, given the already existing infrastructure, the surveillance could rapidly be intensified resulting in a combination of passive and active surveillance components that ensured early detection of SARS-CoV-2 infections in other mink farms. During 2020, the active surveillance components detected new SARS-CoV-2 outbreaks earlier than the official notifications. Furthermore, the active surveillance also detected a fair proportion of mink farms with a subclinical SARS-CoV-2 infection, that might otherwise have been missed or at least have been detected at a later stage.

It is crucial that existing animal health surveillance systems quickly detect emerging zoonotic pathogens that can mutate and potentially jump species to enable swift risk-mitigating actions. These actions are needed to minimize further spread of diseases in the animal population as well as the risk of zoonotic spill-over.

## Figures and Tables

**Table 1:** Available data from the 126 Dutch mink farms from April to November 2020.

Source of the data	Available parameters
Serological ELISA evaluation	Unique herd number (UHI), sample number, date of sampling, date at which the result was available, and serological test result
Early warning RT-PCR testing	UHI, sample number, date of sampling, date at which the result was available, Ct-value, and qualitative result (positive or negative)
Clinical investigation	UHI, date of official diagnosis, farm size, start and end culling date, the surveillance component that indicated the suspicion (screening, early warning, or notification of clinical signs), date first sample was tested positive, whether clinical signs were observed, the date of first signs, and the type of observed clinical signs

**Table 2:** Number of dead mink that were submitted for SARS-CoV-2 RT-PCR testing with their results per farm and per month between June and November 2020.

Month	Number of farms that submitted dead mink ^*∗*^	Median number of submitted mink per farm (range)	Number (%) of farms where SARS-CoV-2 was detected
June	111	3 (1–4)	3 (2.7)
July	103	4 (1–5)	3 (2.9)
August	103	4 (1–14)	20 (19.4)
September	80	15 (1–56)	13 (16.3)
October	65	10 (1–46)	7 (10.8)
November	52	3 (1–18)	2 (3.8)

^*∗*^Farms that did not submit dead mink for the risk-based surveillance were either no longer active or did not report any dead mink. So all eligible farms submitted dead mink.

## Data Availability

Data can be made available upon request after anonymization.
